# Sociodemographic and clinical characteristics of paediatric patients admitted to a neuropsychiatric care hospital in the COVID-19 era

**DOI:** 10.1186/s13052-022-01213-w

**Published:** 2022-02-05

**Authors:** Michela Gatta, Alessia Raffagnato, Federica Mason, Rachele Fasolato, Annalisa Traverso, Silvia Zanato, Marina Miscioscia

**Affiliations:** 1 Child and Adolescent Neuropsychiatric Unit, Department of Women’s and Children’s Health, University Hospital of Padua, 35128 Padua, Italy; 2 Department of Developmental Psychology and Socialisation, University of Padua, 35131 Padua, Italy

**Keywords:** COVID-19, Children, Adolescents, Paediatric age, Mental health, Neuropsychiatry, Hospitalisation

## Abstract

**Background:**

Since the first months of 2020, Italy and the world have been facing the COVID-19 pandemic. In addition to the dangerous and potentially deadly effects on physical health, it has caused a radical change in the lifestyle of the population and a potential danger for mental health too. These events were inserted into the context of a growing epidemiological trend regarding children’s psychiatric disorders in the past decade.

**Aim:**

To study the population of patients admitted to a Neuropsychiatric Hospital Unit of North Italy in the first COVID-19 year, comparing them with the population of patients hospitalised during the year immediately before, according to sociodemographic and clinical variables.

**Methodology:**

The study is an observational retrospective cohort. In total, 198 patients hospitalised due to neuropsychiatric problems from February 2019 to March 2021 were recruited. Data were analysed through mean and standard deviation, t-test, percentages, chi square test, and the Fischer exact test.

**Results:**

Risk factors associated with mental health disorders were similar between the two years. The hospitalisation modality showed a decrease in scheduled hospitalisations compared to urgent ones, and among the reasons that led patients to hospitalisation there was a conspicuous increase in eating disorders. More suicidal and self-harming behaviours occurred in the COVID-19 group too, compared to the previous year. The methods used to attempt suicide were changed considerably, with a prevalence of that attempted within the home. Changes in pharmacological therapies also occurred, necessary for more than 80% of inpatients during the COVID year, with a greater use of neuroleptics. There were alarming data about hospitalisation relapses, which increased from 12.2% in the pre-COVID year to 35.0% in the COVID year.

**Conclusion:**

Data shed light on clinical and policy issues in mental health care during the developmental age. Since the COVID-19 health emergency is not yet over, and its effects, especially on mental health, will be long-term, it is necessary to implement services and activities dedicated to both primary and secondary prevention of neuropsychiatric diseases especially during adolescent ages.

## Background

The COVID-19 epidemic did not only affect physical health conditions, through the disease it directly causes, but it changed the daily life of all people too, invalidating psychological wellbeing due to the contagion containment measures imposed by the government. Unfortunately, to date few studies are available to understand the effects, including long-term ones, of this stressful community event. These studies agree that in the general population there has been a worsening in the quality of life and in perceived wellbeing [1], as well as a greater vulnerability to the negative effects of the COVID-19 pandemic in people who suffer from mental health disorders [2]. A related issue is the alarming number of patients who had impaired mental health assistance during the pandemic, according to findings from different studies conducted in Italy [3], Germany [4], England [5], France [6], India [7], and the United States of America [8]. These studies, on the adult psychiatric population, demonstrated that both outpatient and inpatient settings suffered negative consequences due to the unpreparedness in the COVID-19 disease care logistic and to the onset of social distancing and lockdown measures. Particularly, findings from a recent Brazilian study demonstrate the dramatic decline of consultations and group interventions in mental health and the increase in emergency assistance since the beginning of the COVID-19 pandemic [9]. In line, the WHO survey stated that 93% of all countries have halted mental health services during the COVID-19 pandemic [10]. As a consequence, this phenomenon can aggravate the mental health crisis and generate a parallel pandemic that may last for a long time.

Specifically, valid for childhood, the COVID-19 epidemic affected, to a larger extent, psychological wellbeing versus a smaller effect on physical health [1, 11]. A systematic literature review on data collected in the early period of the pandemic until September 2020, highlighted a deterioration of psychological conditions in young individuals, more markedly in adolescents. Increased depression, anxiety, and other disorders during the COVID-19 pandemic in children and adolescents are reported in review studies [12–15]. Above all, this situation applies to children and adolescents with pre-existing predispositions and to individuals suffering from mental health disorders. Different to the adult population, available studies are either inconsistent or do not estimate the effects of COVID-19 on specific juvenile neuropsychiatric disorders. Particular attention should be paid to eating and self-harming disorders that have been expressed in this pandemic and are among the psychiatric disorders that most often need hospitalisation. Concerning eating disorders, it would seem the COVID-19 pandemic constituted a risk factor for both the onset of and the consequences for the exacerbation of symptomatology [16]. In Italy, according to data of the National Health Institute, eating disorders increased by as much as 37% in the adolescent population [17, 18].

With regard to self-injuring, it seems that the second wave of the COVID-19 pandemic, in particular, has significantly affected suicide rates among children and adolescents [19]. Specifically, months with significantly higher rates of suicide-related behaviours appear to correspond to times when COVID-19–related stressors were heightened [20]. With respect to suicidal behaviours, an observational retrospective study conducted in France from January 2018 to June 2020 found that, during the COVID-19 lockdown (March–May 2020), the number of hospitalisations for suicidal behaviours in children and adolescents decreased by 50% [21]. Another study [22] showed that the incidence rates of suicide-related emergency department encounters among youth in 2020 were comparable with 2019 incidence rates except for a decrease during the lockdown period vs the same period in 2019 and an increase among girls in the second part of 2020 vs the same period in 2019. Youth with no previously documented mental health treatment had more visits in the last trimester of 2020 compared with this period in 2019. In addition, the results in the literature are still exiguous, and presently, it is difficult to make predictions regarding the long-term effects of the pandemic on the mental health of youth and the trend to be expected in admissions to child neuropsychiatry departments; it is clear that the COVID-19 pandemic may worsen mental health for some groups of youth and during different waves of the pandemic. For this reason, it is important to study the phenomenon of hospitalisation since the pandemic outbreak and its ongoing and emergency service–based interventions that may support this population.

### Aim of the study

Within the need for research about the characterisation of pandemic-related neuropsychiatric disorders, the current study aims to describe the population of patients admitted to a Neuropsychiatric Unit of Veneto (North Italy) between February 2019 and March 2021, dividing the sample into two groups: one group composed of patients hospitalised from February 2019 to February 2020 (labelled the “pre-COVID-19 group”), the other group composed by patients hospitalised from March 2020 to March 2021 (labelled the “COVID-19 group”). The purpose of the study is to compare the two groups according to sociodemographic and clinical variables, evaluating the significant changes, in order to pinpoint significant risk factors related to the COVID-19 pandemic for implementing feasible measures in terms of primary and secondary prevention.

## Materials and methods

### Participants

Neuropsychiatric inpatients 0–17 years old hospitalised for more than 24 h in the period between 1 February 2019 and 31 March 2021 were enrolled. Patients who were admitted to a regimen of day hospital were excluded from the study. In total, the inpatients enrolled were 198, 102 hospitalised in the pre-COVID-19 period (February 2019–February 2020) and 96 hospitalised in the “COVID-19 period” (March 2020–March 2021). This last period coincides with the adoption of measures for social isolation and for containment of the COVID-19 epidemic.

### Procedures

The multidisciplinary evaluation carried out during hospitalisation includes neuropsychiatric interviews for the patient and parents; neuropsychological assessment to evaluate the patient’s cognitive and executive functioning; administration of projective tests and structured and/or semi-structured questionnaires; and the observation of family interactions through the Lausanne Trilogue Play (LTP) procedure [23–25].

We conducted an observational retrospective cohort study based on the review of medical materials (clinical records, reports of medical examinations, reports of the neuropsychiatric interviews with the patients and their parents, discharge reports, and clinical reports sent to territorial mental health and social services). Data were treated anonymously; the study was conducted according to the guidelines of the Declaration of Helsinki and approved by the Institutional Ethics Committee of the Padua University Hospital (ref. Protocol 0,031,095, CESC n.25 of 22 April 2021).

Multiple variables, described below, were collected for each patient.

Sociodemographic variables: gender, age at the time of hospitalisation, ethnicity (Caucasian, Latin, African, Asian, or “other” when parents belong to two different ethnic groups), immigration, educational level (primary, middle, or high school), academic or school behavioural problems, peer socialisation (good, difficult, or social withdrawal), bullying or cyber bullying, and family (parents’ marital status, siblings, intra-family problems).

Anamnestic and clinical variables: psychiatric familiarity, chronic pathologies, traumatic life events, previous requests for assistance or previous accesses in neuropsychiatric services, risk behaviour: alcohol and substance use, usage time of devices (more or less than 4 h per day); suicidal self-injury: suicidal ideation, suicide attempt (SA), method of suicidal attempt such as drugs or substances poisoning / self-cutting / other; non-suicidal self-injury (NSSI): frequency of acts (more or less than 5 times per year in accordance with DSM-5 criteria), self-injured body parts, age of the NSSI onset; eating problems.

Hospitalisation related variables: reason for hospitalisation (suicidal and non-suicidal self-injury, anxiety symptoms and/or functional symptoms, eating disorders, psychomotor agitation and/or aggression, psychotic symptoms, and other), hospitalisation modality (urgent or scheduled), way to hospitalisation access (Emergency Department, neuropsychiatric consulting, outpatient service, scheduled hospitalisation, and transfer from another medical unit or from another hospital), duration in days of the hospitalisation, diagnosis according to ICD-10 criteria (F20–29: psychotic disorders, F30–39: affective syndromes, F40–48: neurotic, stress-related and somatoform disorders, F50–59: syndromes and disorders associated with physiological disturbances, F90–98 and F60: syndromes and behavioural disorders with onset usually occurring in childhood/adolescence and personality disorders and other), comorbidity, pharmacological therapy (monotherapy or polytherapy and pharmacological combination: neuroleptics, antidepressants, benzodiazepines, and mood stabilizers), post-discharge services (public and private territorial outpatient care, residential or semi-residential care, intensive hospital monitoring, social services, or family counselling), post-discharge relapse (yes/no and number of readmission to the hospital).

### Statistical analysis

Primarily, descriptive analyses were calculated: mean, standard deviation, t-test for the analysis of continuous variables, percentages, chi-square Test, and the Fischer Test. The statistical significance level was set at *p* < 0.05. Data were analysed using Jamovi Statistic Software.

For some variables (suicide attempt, non-suicidal self-injury, and reason for hospitalisation), in addition to comparison between the pre-COVID-19 year and the COVID-19 year, we also performed a comparison within the COVID-19 group between the first and second wave of contagion (September/October 2020).

## Results

### Sociodemographic and family variables

Sociodemographic and family variables are shown in Table 1.

**Table 1:** Sociodemographic and family variables

	**PRE COVID-19 YEAR**	**COVID-19 YEAR**
HOSPITALISATIONS N	102	96
SEX N (%)	37 M, 65 F (36.3% M, 63.7% F)	33 M, 63, F (34.4% M, 65.6% F)
AVERAGE AGE	13.2 years	13.8 years
CAUCASIAN ETHNICITY N (%)	88 (88.0%)	84 (87.5%)
IMMIGRATION N (%)	9 (8.8%)	5 (5.2%)
HIGH SCHOOL N (%)	44 (45.4%)	49 (51.6%)
MIDDLE SCHOOL N (%)	38 (39.2%)	37 (38.9%)
SOCIALISATION PROBLEMS N (%)	39 (40.2%)	43 (45.7%)
BULLYING VICTIM N (%)	31 (31.0%)	24 (25.5%)
MARRIED/COHABITING PARENTS N (%)	72 (70.6%)	63 (65.6%)
DIVORCED PARENTS N (%)	22 (21.6%)	26 (27.1%)
INTRA-FAMILY PROBLEMS N (%)	43 (43.9%)	31 (34.4%)
1 BROTHER/SISTER N (%)	63 (61.8%)	49 (51.8%)
2 BROTHER/SISTER N (%)	19 (18.6%)	15 (15.8%)
ONLY CHILD N (%)	13 (12.7%)	19 (20.0%)

*M* Male, *F* Female

### Anamnestic-clinical variables and hospitalisation related variables

Recorded predisposing factors for the development of mental disorders were similar in the two groups. Indeed, more than half of inpatients showed psychiatric familiarity; 84% of inpatients of both groups had previously accessed neuropsychiatric services or had received other forms of help (e.g., psychological, psychotherapeutic, and/or psychiatric support); about 40% of inpatients reported at least one traumatic life event; from 38 to 40% of inpatients suffered from one or more chronic pathologies (e.g., asthma, diabetes, allergies, IBD, etc.); 63.4% of inpatients of the pre-COVID-19 group and 69.5% of inpatients of the COVID-19 group had parents or first-degree relatives affected by one or more pathologies (psychiatric familiarity was excluded).

As concerns substances, alcohol and tobacco use, a percentage increase from the pre-COVID-19 year to the COVID-19 year was recorded: respectively from 7.9 to 9.7% for substance use, from 5 to 10.6% for alcohol use, and from 5 to 14.9% for tobacco use. More specifically, the increment of tobacco use was statistically significant (X2 = 5.47, df = 1, *p* = 0.019). Relating to usage time of devices, in the pre-COVID-19 year 40% of inpatients used them less than four hours per day, while 60% of inpatients used them more than four hours per day. In the COVID-19 year the percentages were equally distributed (50% of inpatients used devices less than four hours per day and 50% more than four hours per day).

### Suicidal and non-suicidal self-injury

Regarding suicide attempts, the following differences were detected:

- An increase in suicidal ideation between the pre-COVID-19 year and the COVID-19 year: from 45.1% to 53.8%.
- A decrease in suicide attempts in the COVID-19 era versus the pre-COVID-19 era: from 24.5% to 18.9%.
- A variation in suicidal methods: in the COVID-19 year there was an increment of suicidal attempts through drug or substance poisoning (from 40.0% to 66.7%) and through wrist cutting (from 4.0% to 11.1%) compared to the pre-COVID-19 year; while there was a reduction, from 56.0% to 22.2%, in suicidal methods classified in “other” (defenestration, falling from height, choking, and being hit by fast vehicles).

As concerns suicidality, data between the two waves of the COVID-19 epidemic were compared. The COVID-19 group was, consequently, divided into two subgroups: inpatients hospitalised during the first wave of contagion (from March 2020 to the end of September 2020) and inpatients hospitalised during the second wave of contagion (from October to late March 2021). From this comparison, we observed an increase in suicidal ideation between the first and the second wave (from 50.9% to 57.9%) and an increase in suicide attempts between the first and the second wave (from 14.5% to 25.6%). In order to deeply investigate the changes within the suicidal phenomenon between the two waves of contagion, we evaluated how many inpatients attempted suicide among those with suicidal ideation: they were 28.6% during the first wave and 45.5% during the second wave.

As concerns non-suicidal self-injury (NSSI), the percentage of NSSI inpatients was 36.3% in the pre-COVID-19 year and 37.5% in the COVID-19 year. Relating to NSSI frequency acts, 47.2% of inpatients had an occasional history of NSSI (less than five acts per year) in the COVID-19 year compared to 43.2% of inpatients in the pre-COVID-19 year. Differently, 52.8% versus 56.8% of inpatients had a history of repetitive NSSI (more than five acts per year) respectively in the two years. An opposite trend relating to the number of self-injured body parts (self-cutting was the most common method) was observed: in the pre-COVID-19 year the majority of inpatients self-injured multiple body parts (versus self-injuring a single body part), respectively 60.7% and of 39.3%; during the COVID-19 year there was a reversal of data: 34.4% of inpatients self-injured multiple body parts versus 65.6% of inpatients who self-injured a single body part. This last result was statistically significant (X2 = 4.16, df = 1, *p* = 0,041,). We observed, furthermore, a significant increase in the age of the onset of NSSI from the pre-COVID-19 year (mean age of 12.2) to the COVID-19 year (mean age of 13.8 years).

We performed a comparison between the first wave and second wave of COVID-19 pandemic on non-suicidal self-injury, observing a statistically significant difference: self-injured inpatients in the first wave were 29.1% compared to 48.8% in the second wave (X2 = 3.89, df = 1, *p* = 0,049).

### Modality and reason for hospitalisation

The hospitalisation modality was primarily classified as either urgent or scheduled. The results showed a decrease in scheduled hospitalisations from 12.7% in the pre-COVID-19 year to 6.3% in the COVID-19 year and an increase in urgent hospitalisations from 87.3% to 93.8%. A statistically significant difference was found (X2 = 14.2, df = 4, *p* = 0,007) comparing the path to accessing hospitalisation. A decrease in all ways to access was found, except for hospitalisation following a visit to the outpatient service of the hospital (Table 2). Moreover, the investigation of the reasons for hospitalisation showed differences between the two years and between the first and second wave regarding eating disorders, psychomotor agitation, and aggression (Table 3).

**Table 2:** Modality of access to hospitalisation in the pre-COVID-19 year and the COVID-19 year

	ED N (%)	NPI CONSULTING N (%)	OUTPATIENT EXAMINATION N (%)	SCHEDULED N (%)	TRANSFER N (%)
PRE-COVID-19	**52 (51.0%)**	2 (2.0%)	**6 (5.9%)**	13 (12.7%)	29 (28.4%)
COVID-19	**42 (43.7%)**	1 (1.0%)	**23 (24.0%)**	6 (6.3%)	24 (25.0%)

*ED* Emergency Department, *NPI* Neuropsychiatric

*p value* = *0.007*

**Table 3:** Reasons for hospitalisation: pre-COVID-19 year and COVID-19 year, I and II wave of contagion

	SUICIDAL AND NON-SUICIDAL SELF- INJURY N (%)	ANXIETY AND/OR FUNCTIONAL SYMPTOMS N (%)	EATING DISORDERS N (%)	PSYCHOMOTOR AGITATION/ AGGRESSION N (%)	PSYCHOTIC SYMPTOMS N (%)	OTHER N (%)
PRE-COVID-19	30 (29.4%)	14 (13.7%)	**12 (11.8%)**	17 (16.7%)	**10 (9.8%)**	10 (18.6%)
COVID-19	32 (33.7%)	14 (14.7%)	**18 (18.8%)**	16 (16.8%)	**4 (4.2%)**	12 (11.6%)
I WAVE	17 (30.9%)	9 (16.4%)	**5 (9.1%)**	**12 (21.8%)**	3 (5.5%)	9 (16.4%)
II WAVE	15 (36.6%)	5 (16.2%)	**13 (31.7%)**	**4 (9.8%)**	1 (2.4%)	3 (7.3%)

*p value (pre and COVID-19 years)* = *0.330*

*p value (I and II wave)* = *0.051*

### Diagnosis, therapeutic indications, and post-discharge relapse

In Table 4, *(at the end of the manuscript)* data on diagnosis and discharge are reported, relating both to the first and second ICD 10 diagnosis.

**Table 4:** First and second ICD-10 diagnosis

***FIRST DIAGNOSIS***	***ICD-10 CODE***	***PRE-COVID-19*** **N (%)**	***COVID-19*** **N (%)**
AFFECTIVE SYNDROME	F30-F39	31 (36.0%)	29 (31.9%)
NEUROTIC, STRESS-RELATED, AND SOMATOFORM DISORDERS	F40-F48	28 (31.5%)	27 (29.7%)
DISORDERS ASSOCIATED WITH PHYSIOLOGICAL DISTURBANCES	F50-F59	**9 (10.1%)**	**14 (15.4%)**
PERSONALITY DISORDERS, SYNDROMES, AND BEHAVIOURAL DISORDERS WITH ONSET USUALLY OCCURRING IN CHILDHOOD/ADOLESCENCE	F60, F90-F98	10 (11.2%)	11 (12.1%)
PSYCHOTIC DISORDERS	F20-F29	7 (7.9%)	5 (5.5%)
OTHER	OTHER CODES	3 (3.4%)	5 (5.5%)
***SECOND DIAGNOSIS***	***ICD-10 CODE***	***PRE-COVID-19*** **N (%)**	***COVID-19*** **N (%)**
AFFECTIVE SYNDROME	F30-F39	9 (14.5%)	11 (15.7%)
NEUROTIC, STRESS-RELATED, AND SOMATOFORM DISORDERS	F40-F48	22 (35.5%)	29 (41.4%)
DISORDERS ASSOCIATED WITH PHYSIOLOGICAL DISTURBANCES	F50-F59	1 (1.6%)	2 (2.9%)
PERSONALITY DISORDERS, SYNDROMES, AND BEHAVIOURAL DISORDERS WITH ONSET USUALLY OCCURRING IN CHILDHOOD/ADOLESCENCE	F60, F90-F98	16 (25.8%)	18 (25.7%)
PSYCHOTIC DISORDERS	F20-F29	1 (1.6%)	0 (0.0%)
OTHER	OTHER CODES	13 (21.0%)	10 (14.3%)

*ICD-10* International Classification of Disease, X edition

*p value (I diagnosis)* = *0.833, p value (II diagnosis)* = *0.722*

For comorbidity (presence of two or more diagnoses in the same patient), we observed an increase from 71.1% of inpatients with at least one comorbidity in the pre-COVID-19 year, to 77.3% in the COVID-19 year. In total, 18 inpatients did not receive a complete diagnosis (early discharge due to parent choice or transfer to another medical unit). Hospitalisation lasted an average of 20.1 days in the pre-COVID-10 year and an average of 18.1 days in the COVID-19 year.

**Fig. 1:**
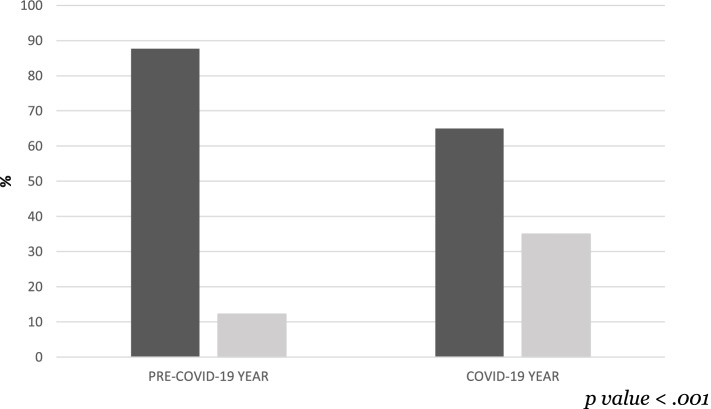
Percentages of patients with post-discharge relapse between the two years

Data on post-discharge hospital readmission show that in the pre-COVID-19 year the relapses were 12.2%, while in the COVID-19 year this percentage rose to 35.0%. This result means that in the COVID-19 year, compared to the pre-COVID-19 year, more inpatients were already hospitalised at least once (Fig. 1).

The columns in dark grey represent the percentage of patients at first hospitalisation; the columns in light grey represent the percentage of patients with post-discharge relapse.

Regarding the therapeutic plan at the moment of discharge, psychotherapy was suggested to 84.1% of pre-COVID-19 inpatients and to 75.5% of COVID-19 inpatients. A slight opposite trend was registered about pharmacotherapy (81.4% of pre-COVID-19 inpatients and 88.4% of COVID-19 inpatients). The percentages of inpatients with pharmacological polytherapy compared to monotherapy were unaltered (77% of inpatients in both groups), while a statistically significant difference (X2 = 17.6, df = 8, *p* = 0.025) was found for neuroleptic association with other pharmaceuticals (from 44% pre-COVID-19 year to 61% in the COVID-19 year) and antidepressants in monotherapy (from 2% to 5.3%).

Statistically significant differences between the two years (X2 = 14.0, df = 5, *p* = 0,015) were also found in post-discharge admission to territorial mental health services: territorial outpatient treatments decreased both in public and in private services (from 70% in the pre-COVID-19 year to 46% in the COVID-19 year); an increase from 20% in the pre-COVID-19 year to 29% in the COVID-19 year was observed for residential and semi-residential care (e.g., residential therapeutic centres, daily centres, and eating disorders centres); an increase in intensive monitoring interventions by hospital and by social and family services (from 10% in the pre-COVID-19 year to 25% in the COVID-19 year) was also registered.

The number of hospitalisations is reported in Table 5, which shows an increase in the number of post-discharge relapses from the pre-COVID to COVID period.

**Table 5:** Number of hospitalisations in two years

	1° H N(%)	2° H N(%)	3° H N(%)	4° H N(%)	5° H N(%)	6° H N(%)
PRE-COVID-19	73 (89.0%)	**6 (7.3%)**	2 (2.4%)	**1 (1.2%)**	**0 (0.0%)**	**0 (0.0%)**
COVID-19	52 (65.0%)	**14 (17.5%)**	2 (2.5%)	**7 (8.8%)**	**4 (5.0%)**	**1 (1.3%)**

*H* Hospitalisation

*p value* = *0.006*

## Discussion

The epidemiological trend of neuropsychiatric disorders has been constantly growing in the last decade [26, 27]. A new social and sanitary scenario due to SARS-CoV-2 infection outbreak was inserted in the context of this alarming trend. Social distancing, numerous limitations, changes in daily routine, and a sense of anxiety and uncertainty for health and for the future affected negatively the psychological wellbeing of both adults and children. In the field of mental health, this study aimed to observe the way in which the COVID-19 sanitary emergency affected the clinical features and the management of patients hospitalised for neuropsychiatric problems, comparing the first COVID year with the previous one.

Patients appeared generally similar regarding sociodemographic and family variables. The majority of inpatients were female adolescents between 12 and 17 years. This result is widely documented by the literature, according to which before puberty males and females presented the same rate of mental illness, while this rate rises for the female sex in adolescence [28, 29]. According to the fact that more than half of inpatients were adolescents, it was observed that almost half of them attended high school, while a quarter of them attended middle school. It should be kept in mind that high school students suffered more from containment measures (e.g., suspension of the educational offline activities for longer periods). In this sense, different studies questioned the school closure’s efficacy as anti-contagion measure, assuming that the damages, at a psychological level, for children and adolescents, will exceed over time the benefits related to contagion [30, 31].

Data related to bullying, peer socialisation, and time use of devices were similar in the pre-COVID and COVID samples. As we know from the literature, there was a strong association between being a victim of bullying and psychosocial functioning impairment, as well as physical and psychopathological symptoms [32–34]. Similarly, the association between excessive internet use (more than three hours per day), already increased in the pre-COVID era [35, 36], and internalising mental health problems (specifically anxiety, depression, and mixed mental disorders) is well known [37]. Our data, which did not show substantial differences between the two years, should be interpreted in the light of psychopathology symptoms, which are themselves associated with the abovementioned factors (difficult socialisation, bullying, and internet addiction) [38].

Regarding the use of substances, such as alcohol and tobacco, an increase was observed, especially for alcohol and tobacco. This result could be controversial: on one hand, mental health disease due to forced social distancing and other COVID 19 related limitations and changes could facilitate increase in alcohol, other substances, and tobacco consumption, and, on the other hand during the most restrictive period of COVID-19 epidemic in which it was forbidden to move without demonstrable need, the availability of these substances was limited, especially for adolescents. In any case, there was a stronger association between emotional–behavioural vulnerability and substance abuse, from adolescence to adulthood [39]. Data reported by Italian National Health Institute during the COVID-19 epidemic, show a change in the consumption typology and modality: on one side, a reduction in substances consumed, usually a stimulant (amphetamine and/or cocaine) that in the pre-COVID-19 era could be consumed in social contexts (e.g., café, discotheque, etc.,) and, on the other side, an increase in substances consumed with a calming effect (opioid, benzodiazepines, and cannabinoid). Moreover, the COVID-19 group of inpatients showed an increase in tobacco smoking three times more than the pre-COVID-19 group. An Italian study on the adult population highlighted that tobacco smokers increased by 9.1% from 27 April to 3 May 2020. [40]. In terms of other vulnerability factors on the development of psychiatric pathologies, the results were similar for both years: the majority of inpatients had psychiatric familiarity, had parents or first-degree relatives who suffered from another pathology, had previously accessed neuropsychiatric services or requested other forms of assistance (e.g., psychological, psychotherapeutic, or psychiatric support). About 40% of inpatients reported at least one traumatic life event and were also affected by one or more non psychic chronic pathologies.

Special attention was drawn to suicidal and non-suicidal self-injury, because of the increase in self-harming related accesses to mental health services, as detected in clinical daily practice and in the scientific literature [41]. COVID-19 year inpatients reported more suicidal ideation than pre-COVID-19 era inpatients; nevertheless, inpatients of the COVID-19 group attempted suicide more or less with the same frequently as the pre-COVID-19 inpatients. Among COVID-19 year patients with a suicide attempt, poisoning and wrist-cutting were increased compared to other suicidal methods. Literature relating to COVID-19 epidemic effects on suicidal behaviour, although limited to date, highlighted a significant increase in both suicidal ideation and attempts among adolescents in the COVID-19 period compared to the previous year, specifying that rates of suicide ideation and attempts were higher during some months of 2020 as compared with 2019 but were not universally higher across this period [19]. Our data showed a larger suicidal ideation increase than suicidal attempts, suggesting the possibility of higher suicide rates in the future. According to that, a recent first meta-analysis estimate of suicidal ideation based on a large sample from different countries and populations has shown the rate of suicidal ideations during COVID-19 pandemic was higher than that reported in studies on the general population prior to the pandemic [20]. The increase in suicidal ideation in the COVID-19 year is coherent with the rise of anxiety, stress, and depression, related to the protracted period of lockdown and the interruption of educational and sport activities, known risk factors for the development of self-injury and suicidal behaviours [42, 43]. The relative reduction in suicide attempts could be due to limiting/ protective actions of the lockdown on children and adolescents, who were more supervised by parents. In line, it is the variation of percentage related to suicidal methods in the COVID-19 year: substance and drug poisoning and wrist-cutting were reported as the more feasible methods in the domestic context. When globally reading this data, it must be also said that the absence of significant differences in the percentages of suicidal ideation and suicide attempts between two years is consistent with the trend of these behaviours already rising before the COVID-19 epidemic. Indeed, a study conducted in the U.S.A. had already shown an alarming growth of hospitalisations for suicidal ideation and for suicide attempts among children and adolescents from 2008 to 2015, where the percentage was almost doubled between the first and the last year [44]. Even in Italy this phenomenon occurred in some hospitals of both central and north areas [45].

We examined the suicidality phenomena within the hospitalised patients’ sample during the COVID-19 epidemic, in order to verify any change between the first wave (from March 2020 to September 2020) and the second wave (from October 2020 to March 2021). Results showed a slight increase in suicidal ideation (from 50.9% to 57.8%) and a more marked increment of suicide attempts (from 14.5% to 25.6%) from the first to the second wave. Comparing percentages of suicide attempts among patients with suicidal ideation, we observed a greater increase along the two years (from 28.6% to 45.5%). This result is consistent with a Japanese study that reported a reduction of 14% in suicide rates during the first five months of epidemic and an increase of 16% in suicide rates in the subsequent four months, especially among females (37%) and children and adolescents (49%) [46]. We then suppose that the lower rate of suicide attempts during the first wave could depend not only on major monitoring by the parents but even more on the temporary reduction in social and performance stress burden linked to school closure. Moreover, it should be kept in mind that in Italy a special Decree-Law was approved for the academic year 2019–2020 in order to promote the inclusion of the more vulnerable subjects, which facilitated the transition to higher class for students with poor academic performance (Decree-Law 8 April 2020, n. 22). This measure certainly reduced school related anxiety and stress in many adolescents. On the contrary, the increase in suicide attempts during the second wave of the epidemic could be mainly induced by what is called “back-to-school stress”. Returning to school with new rhythms and rules, in addition to social and test anxiety in many students, together with the uncertainty due to constant routine changes caused by contagion curve raising, could have a *rebound* effect on the psychological state of young people.

As for what concerns non-suicidal self-injury, this occurred in more than one third of inpatients and, among these, more than half self-injured more than five times. We observed an opposite trend for the number of self-injured body parts, which decreased in the COVID year compared with the previous period.

This result could be related to a greater possibility of child monitoring by caregivers, thanks to lockdown and social restrictions. A significant raising of the NSSI onset age was registered, from a mean of 12.2 years in the pre-COVID-19 year to a mean of 13.8 years in the COVID-19 year. These data, which are different from the trend reported by literature about a reduction in the NSSI onset’s mean age in adolescents [47, 48], could be linked to teenagers’ greater suffering because of major restriction measures compared to younger subjects during the COVID-19 pandemic.

Also concerning non-suicidal self-injury, we observed a significant increase from the first (29.1%) to the second wave (48.8%), which was in line with the literature that highlighted an increase in NSSI behaviours in adolescents during the COVID-19 pandemic compared to the pre-COVID-19 era [49]. Other studies reported a “wave trend”, with an initial decrease in self-harming during the first months of COVID-19 pandemic, followed by a strong rise during subsequent months [50].

We classified hospitalisation modality into urgent and scheduled, showing a reduction in scheduled hospitalisations from 12.7% in the pre-COVID-19 year to 6.3% in the COVID-19 year, and, on the contrary, an increase in urgent hospitalisations. This result is in accordance with the generalised interruption of scheduled hospitalisations at the peak of COVID-19 epidemic due to institutional rules on one side and to the increase in COVID-19 issue-related acute psychiatric conditions on the other side. Interesting differences emerged by comparing the different paths to access to hospitalisation. In particular, we saw an increase in hospital admission following a visit to our outpatient service (from 5.9% to 24.0%) and a reduction in hospitalisation following Emergency Department access (from 51.0% in the pre-COVID-19 year to 43.8% in the COVID-19 year). These results are similar to those obtained from a study conducted in the Emergency Department (ED) of two of Modena’s hospitals, which compared urgent ED psychiatric consultations during the first six months of the COVID-19 pandemic with ones during the corresponding period of the previous year. The authors found a general reduction in ED consultations, probably due to contagion fear and to lockdown-related rules [51]. The increased percentage in our hospitalisation admissions after an outpatient examination could be explained by both the interruption of scheduled recoveries and the restriction imposed to territorial mental health services, so that patients were referred to outpatient service at a hospital. Moreover, Modena’s study recorded an increase in care requests by those people who already suffered from psychiatric disorders and/or were already treated by psychiatric territorial services, compared to 2019.

As regard to reasons for hospitalisation, we recorded two main changes: the eating disorders’ increase (from 11.8% in the pre-COVID-19 year to 18.9% COVID-19 year) and the psychotic symptoms’ decrease (from 9.8% to 4.2%). The analysis between the first and the second COVID-19 pandemic wave, showed that hospitalisations for eating disorders more than tripled (from 9.1% to 31.7%), as well as hospitalisations for agitation and aggression. The rise in eating disorders is in accordance with findings from other international studies; in particular, an Australian one recorded an increment of restrictive and binge eating behaviours in the general population, which were associated with compensatory physical activities among those people who previously suffered from eating disorders [52]. A study conducted in Italy and France, describing the ways in which COVID-19 pandemic affected the risk for eating disorder development, emphasised both the increase in risk factors and the decrease in protective factors related to COVID-19 epidemic’s consequences (daily routine interruption, outdoor activities limitations, greater exposure to social media, special diets with health related intents, social distancing, greater difficulty in the access to care, and emotion regulation difficulties related to uncertainty and to contagion fear) [53].

As concerns the reduction in hospitalisations because of psychotic symptoms, we suppose the lockdown could have constituted a protective factor for children who suffer from this disorder. The smaller exposure to external stimuli and the constant care by a caregiver could create a protective context for these patients, preventing the exacerbation of a psychotic crisis, specifically paranoid type ones. The differences about ICD-10 diagnosis between the two considered periods are in accordance with the abovementioned findings related to reasons for hospitalisation. Specifically, we observed an increase in IC10 F50-F59 codes among first diagnoses, which include diagnoses related to eating disorders.

One of the most important differences concerns post-discharge relapses. The number of inpatients who were hospitalised again after the first discharge almost tripled during the COVID-19 year. This result indicates that more patients were already hospitalised at least once during the COVID-19 year. Moreover, in the pre-COVID-19 year, 89% of patients were hospitalised for the first time (no post-discharge relapse), while during COVID-19 year this percentage lowered to 65%. Differently, the percentage of patients with two hospitalisations doubled from 7.3% in the pre-COVID-19 era to 17.5% in the COVID-19 year; patients with four hospitalisations were 1.2% in the pre-COVID-19 year, while they increased to 8.8% in the COVID-19 year; patients with five or six hospitalisations were registered only during the COVID-19 year. These data agree with multiple studies, one of those conducted by World Health Organization (WHO), that emphasised COVID-19 pandemic as a risk factor for multiple psychiatric disorders’ exacerbation and relapses [2].

Moreover, other studies conducted in Italy and Spain are consistent with our findings, corroborating the hypothesis that the greater number of relapses could be explained by limitations imposed on neuropsychiatric territorial services, unable to guarantee the continuity of mental health assistance, and by the increase in psycho-behaviour distress in children and adolescents who already suffered from psychiatric disorder and who were particularly affected by the pandemic. [54, 55]. In fact, the paradox of the territorial clinical-assistance management of psychiatric patients was highlighted: services operating at a lower rate, precisely in a historical context in which the most vulnerable people were subjected to greater stress and therefore needing care.

Those aspects influenced the hospital and the high-intensive therapeutic residential structures’ burden, as well as the greater use of psychiatric drugs. In fact, we suppose that these last findings are related to the increase in psychiatric symptoms’ severity to a first extent and to the reduction in psychological and psycho-educational treatment availability to a lesser extent. Specifically, the greater use of neuroleptics correlates with the management of behavioural and emotional dysregulation disorders, frequently associated with self-harming conduct and more severe eating disorders, in particular, anorexia nervosa [56, 57]. Suicidal and non-suicidal dimensions along with eating disorders, then, were the most increased problems among inpatients during COVID-19 epidemic and, accordingly, we observed an increment of post-discharge access to residential care structure. Current studies seem to agree on the fact that the SAR COVID 2 infection outbreak constituted a predisposing factor for the development and exacerbation of eating disorders, which were associated with a quantitative increment of mental health service patients [5].

### Limits of the study

Our study presented some limitations related to missing data and to patients’ heterogeneity in age and other correlated variables, due to the retrospective design. Another limitation is related to the limited sample size and the statistical comparison carried out between closed time periods (2019–2020 and 2020–2021). Moreover, the SARS-Cov-2 infection sanitary emergency is still ongoing, and for this reason it was not yet possible to obtain a complete overview of its effects.

## Conclusion

The epidemiology of psychiatric disorders at the developmental age has been rising for years both in a national and international context. Within this scenario, the COVID-19 epidemic has emerged, with detrimental effects on mental health in all categories of individuals, although children and adolescents seem to be affected more. This study aimed to investigate, through a comparison between the pre-COVID-19 years and the first COVID-19 epidemic year, what sociodemographic and clinical changes were related to the COVID-19 pandemic in the psychiatric population hospitalised at the Neuropsychiatric Unit of Veneto, a regional centre specifically dedicated to psychiatric disorders, the only one which deals also with emergency clinical conditions. Our preliminary findings, where no data about psychiatric hospitalisations in developmental age at the time of COVID-19 pandemic are available to date, highlighted multiple information useful to develop reflections on both clinical-assistance and sanitary-organisational issues. Particularly, there emerged a prevalence of psychopathologic problems related to self-injury and eating disorders; the need for multimodal treatments (e.g., psychological, educational, rehabilitative, neuropsychiatric, and psychopharmacological treatment); the shortage of territorial socio-sanitary services for post-discharge treatments; and the increased severity of psychopathological problems that required rehospitalisation and high-intensive care treatments.

These results, in the context of the SARS-Cov-2 pandemic, which is still ongoing and its consequences will remain, should be key points for reflection and decision about awareness-raising actions related to mental health problems in the juvenile population; for medical staff training and updating on evidence-based treatments, with particular regard to psychopharmacological practice (which is still little known and used not enough by territorial professionals); for the support of socio-sanitary policies towards territorial developmental age neuropsychiatric and psychological services, considering the limitations of telemedicine and the growing demand for the access to mental health care. It certainly is worthwhile to continue the work of collecting data relating to the COVID-19 pandemic period to verify step by step the trend of psychopathology in the various pandemic phases. Taking action to improve and implement neuropsychiatric services will be fundamental for limiting the epidemic effect on the burden of juvenile mental health disorders, keeping in mind that today’s children and adolescents will be tomorrow’s adults.

## Data Availability

The datasets used and/or analysed during the current study are available from the corresponding author on reasonable request.

## Abbreviations

COVID-19: Corona Virus Disease 2019
df: Degrees of freedom
ED: Emergency Department
H: Hospitalisation
ICD-10: International Classification of Disease, X edition
NPI: Neuropsychiatric
NSSI: Non-Suicidal Self-Injury
SA: Suicide Attempt
USA: United States of America
WHO: World Health Organization
